# Corticospinal control from M1 and PMv areas on inhibitory cervical propriospinal neurons in humans

**DOI:** 10.14814/phy2.13387

**Published:** 2017-10-30

**Authors:** Louis‐Solal Giboin, Sina Sangari, Alexandra Lackmy‐Vallée, Arnaud Messé, Pascale Pradat‐Diehl, Véronique Marchand‐Pauvert

**Affiliations:** ^1^ Sensorimotor Performance Lab Sport Science Department Universität Konstanz Konstanz Germany; ^2^ Sorbonne Universités Laboratoire d'Imagerie Biomédicale (LIB) UPMC Univ Paris 06, INSERM, CNRS Paris France; ^3^ Department of Computational Neuroscience University Medical Center Eppendorf Hamburg University Hamburg Germany; ^4^ Département des maladies du système nerveux AP‐HP Hôpital Pitié‐Salpêtrière Paris France

**Keywords:** Corticospinal tract, Humans, Motor cortex, Premotor cortex, Propriospinal neurons, TMS

## Abstract

Inhibitory propriospinal neurons with diffuse projections onto upper limb motoneurons have been revealed in humans using peripheral nerve stimulation. This system is supposed to mediate descending inhibition to motoneurons, to prevent unwilling muscle activity. However, the corticospinal control onto inhibitory propriospinal neurons has never been investigated so far in humans. We addressed the question whether inhibitory cervical propriospinal neurons receive corticospinal inputs from primary motor (M1) and ventral premotor areas (PMv) using spatial facilitation method. We have stimulated M1 or PMv using transcranial magnetic stimulation (TMS) and/or median nerve whose afferents are known to activate inhibitory propriospinal neurons. Potential input convergence was evaluated by studying the change in monosynaptic reflexes produced in wrist extensor electromyogram (EMG) after isolated and combined stimuli in 17 healthy subjects. Then, to determine whether PMv controlled propriospinal neurons directly or through PMv‐M1 interaction, we tested the connectivity between PMv and propriospinal neurons after a functional disruption of M1 produced by paired continuous theta burst stimulation (cTBS). TMS over M1 or PMv produced reflex inhibition significantly stronger on combined stimulations, compared to the algebraic sum of effects induced by isolated stimuli. The extra‐inhibition induced by PMv stimulation remained even after cTBS which depressed M1 excitability. The extra‐inhibition suggests the existence of input convergence between peripheral afferents and corticospinal inputs onto inhibitory propriospinal neurons. Our results support the existence of direct descending influence from M1 and PMv onto inhibitory propriospinal neurons in humans, possibly though direct corticospinal or via reticulospinal inputs.

## Introduction

In humans, the C3–C4 propriospinal system constitutes a complex spinal circuitry, including excitatory interneurons with long intraspinal and diffuse projections to upper limb motoneurons. The synaptic transmission through excitatory propriospinal neurons is modulated by feedback and feed‐forward inhibitory interneurons, all receiving peripheral and descending inputs. This system can thus integrate sensory feedback and corticospinal inputs from the primary motor cortex (M1) to regulate the motor command en route to the motoneurons and to control the motoneuron activity according to movement conditions (Pierrot‐Deseilligny and Burke 2012). This system has been shown to be particularly involved in reach‐to‐grasp and digit movements in primates and humans (Iglesias et al. 2007; Giboin et al. 2012; Isa et al. 2013). We found evidences for inhibitory propriospinal neurons in humans, as described in cats (Alstermark et al. 1984a,b), but this inhibitory system has been investigated to a lesser extent than the excitatory one (Lourenço et al. 2007b; Marchand‐Pauvert and Iglesias 2008). To date, the corticospinal control onto the inhibitory propriospinal system has never been investigated in humans.

Inhibition of spinal motoneurons mediated by putative inhibitory propriospinal neurons in humans, has been revealed using peripheral nerve stimulation only (Lourenço et al. 2007b), as performed at first for the excitatory system (Malmgren and Pierrot‐Deseilligny 1987, 1988a,b). Since then, the propriospinal inhibition has been shown to be reduced in patients with writers' cramp, and this has been attributed to altered descending influence on spinal circuitry (Lourenço et al. 2007a). Therefore, we assumed that the inhibitory propriospinal system would participate in the selective descending activation of relevant motoneurons during movements and would help to prevent unwilling muscle activation. In the same way, feed‐forward and feedback inhibitory control of excitatory propriospinal system can participate in the tune control of upper limb movements by disfacilitating the descending motor command to inappropriate motoneurons. Such a system has the advantage to select the relevant motoneurons during movement, without hyperpolarizing the other ones, which thus stay free for discharging in case of rapid adjustments during unexpected motor adaptations (Pierrot‐Deseilligny and Burke 2012). Due to the powerful feed‐forward and feedback inhibitory control of excitatory propriospinal neurons (Alstermark et al. 1999; Nicolas et al. 2001), it has been suggested that pyramidal tract neurons (PTNs) in the ventral premotor cortex (PMv) would prevent movement during observation‐based motor learning through their possible projections onto the feed‐forward inhibitory interneurons limiting the descending propriospinal excitation to motoneurons (Kraskov et al. 2009). A direct inhibition, hyperpolarizing motoneurons would be more efficient to prevent muscle activation under such conditions.

We therefore addressed the question whether inhibitory propriospinal neurons in humans receive corticospinal inputs from the primary motor cortex (M1) and from PMv. For this, we have used the spatial facilitation method (Eccles and Lundberg 1957), which was first adapted in humans to further support the corticospinal control onto excitatory propriospinal neurons (Pauvert et al. 1998). This method consists in stimulating a well‐known projection to the target interneurons and a hypothesized projection with a timing allowing a theoretical convergence of the two inputs on a common interneuron pool. Comparing the summation of effects on motoneuron excitability after isolated stimuli and those produced on combined stimuli, allows demonstrating the existence of spatial summation at interneuron level and therefore common projections on target interneurons. Thus, we have stimulated M1 or PMv with a subthreshold transcranial magnetic stimulation (TMS) and the median nerve at the wrist level with a percutaneous electrical stimulation. The latter is known to activate particularly inhibitory propriospinal neurons (Lourenço et al. 2007b). We studied the effects of stimulation conditions (isolated vs. combined stimuli) on motoneuron excitability of wrist extensor muscle assessed by investigating the changes in reflex amplitude. Finally, to give further evidence for a direct corticospinal control from PMv, bypassing M1, the effect of TMS over PMv have been investigated before and after paired continuous theta burst stimulation (cTBS) applied over M1, which is known to inhibit M1 excitability (Goldsworthy et al. 2012).

## Methods

### Participants and ethical approval

The participants gave informed written consent to the experimental procedures before participation to the study, which were approved by the ethics committee of the Pitié‐Salpêtrière Hospital (CPP Ile de France VI). Subjects' consent and study procedures conformed to the standards set by the Declaration of Helsinki.

### EMG recordings

The subjects were sitting on a chair with the palmar side of the hand and the forearm resting on a table; the joint angle at elbow level was about 50° and at shoulder level about 10°. Electromyographic (EMG) activity was recorded with bipolar surface electrodes (foam disposable electrodes with solid gel, 2‐cm apart; FIAB, Florence, Italy) placed over the muscle belly of extensor carpi radialis (ECR), abductor pollicis brevis (APB) and first dorsal interosseous (FDI). Electromyogram activity was amplified (×2.000–10.000) and filtered (0.1–1‐kHz bandpass; D360 8‐Channel Patient Amplifier, Digitimer Ltd, Hertfordshire, UK) before being digitally stored on a personal computer (2‐kHz sampling rate; Power 1401 and Signal Software, CED, Cambridge, UK). The mean level of ECR EMG activity produced during the maximal voluntary contraction (MVC) during tonic wrist extension was evaluated in each participant. Then, all EMG recordings were performed during weak tonic wrist extension with ECR EMG, below 5% EMG developed at MVC.

### Stimulation

#### Mechanical stimulation

Tendon taps were applied to the distal part of the tendon of ECR with an electromagnetic hammer placed on the dorsal aspect of the hand. Mechanical stimuli produced fast transient muscle stretches (8 mm during 5 ms), which elicited afferent volleys in group Ia fibers that activated myotatic reflex. The resulting compound muscle action potential in ECR EMG, or tendon reflex (T reflex), occurred at 16.5–23 ms (mean 19.7 ± 0.6 ms).

#### Peripheral stimulation

Median nerve was stimulated (1‐ms rectangular electrical stimulation; DS7A, Digitimer Ltd, Hertfordshire, UK) at the wrist level using bipolar surface electrodes (0.5 cm^2^, 1‐cm apart). The motor threshold (MT) was determined according to the occurrence of clinical muscle twitch and motor (M) response in APB EMG, and the intensity of conditioned pulses was adjusted at 2 × MT. The radial nerve was stimulated using two 5‐cm^2^ brass electrode placed on the dorsal aspect of the arm with cathode proximal to the spinal cord; the electrodes were positioned to produce a twitch in ECR assessed by tendon palpation. The intensity was increased to evoke the maximal M response (Mmax) in ECR EMG, and then it was decreased to produce a sizeable Hoffmann reflex (H reflex; mean latency 19.2 ± 1.1 ms, range 15.5–22 ms).

#### Transcranial magnetic stimulation

TMS was delivered using a Magstim Rapid (Magstim Co Ltd., Whitland, UK) and a figure‐of‐eight coil positioned so as to produce current field in the postero‐anterior direction. The participants wore a cap on which the position of the coil was marked to ensure the stability of TMS across the experiment. First, the coil was positioned over the primary motor area (M1), at the optimal site (hot spot) for producing a motor evoked potential (MEP) in the contralateral ECR EMG during tonic contraction (EMG <5% EMG at MVC). The active motor threshold (AMT) was determined as the minimum intensity to elicit an MEP in ECR EMG ≥50 μV in 5 out of 10 consecutive single TMS pulses during tonic ECR contraction with EMG mean level <5% EMG at MVC. Secondly, the hot spot for evoking an MEP in FDI EMG was also determined and the coil was positioned 3 cm anteriorly and 2.5 cm laterally to stimulate the ventral premotor area (PMv) (Bäumer et al. 2009).

### Experimental protocols

#### Median‐induced ECR MEP suppression

Preliminary experiments were first performed in 6 subjects (4 females, age range 22–38 years old, mean age 28.6 ± 2.7 years old); all were right‐handed (Oldfield 1971). These experiments were undertaken to determine the optimal interstimulus intervals (ISIs) between median nerve stimulation and TMS over M1 for interaction between peripheral and descending inputs at spinal level. The results were used to develop the main protocol of the study (see below). For the preliminary experiments, TMS was delivered over M1 and its intensity was set so as to produce an MEP of ~10% Mmax in ECR EMG during tonic ECR contraction with ECR EMG <5% EMG at MVC (71.0 ± 4.4% MSO, 60–88% MSO depending on the subject, which corresponded to 120–130% AMT). This size of MEP corresponds to a MEP within the linear part of the recruitment curve (around I50, that is the TMS intensity at which the MEP is half its maximal MEP size) where the corticospinal system is very sensitive to conditioning response. Twenty TMS alone and 20 TMS combined to median nerve stimuli (2 × MT) were randomly alternated (0.6 Hz) with ISIs between 0 and 13 ms (1‐ms step); median nerve stimuli being delivered before TMS to optimize the convergence of peripheral and corticospinal volleys at spinal level.

#### Effect of combined median nerve stimuli and TMS on ECR reflex

**Figure 1:**
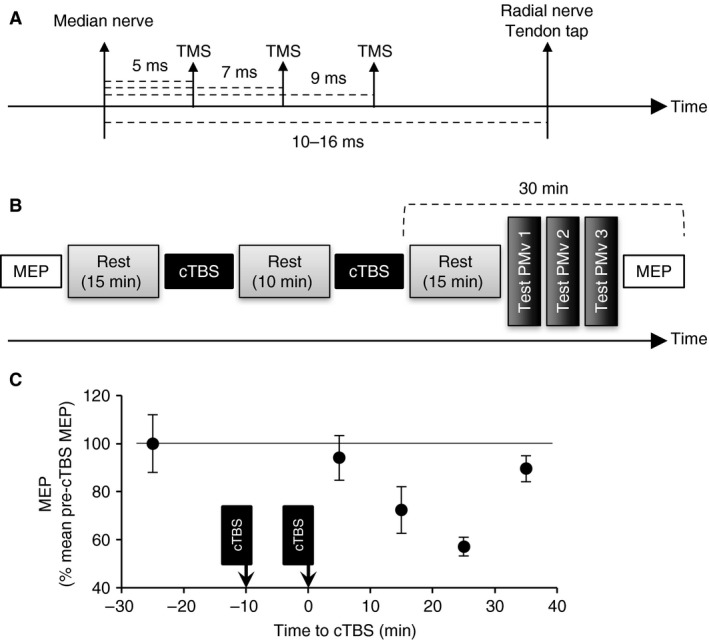
Experimental designs. (A) the stimulation sequence for triple stimulation protocol includes median nerve stimuli delivered between 10 ms and 16 ms (depending on the subject) before stimuli producing reflexes in ECR EMG (tendon tap or radial nerve stimulation), and TMS which was delivered 5, 7 or 9 ms after median nerve stimuli. TMS was applied over M1 or PMv. (B), the protocol using cTBS started with evaluation of MEP size produced in ECR EMG by TMS over M1 at 1.5 × AMT, followed by 15 min rest. After this first rest, the first cTBS was applied over M1 (intensity 0.7 × AMT, 3 pulses at 50 Hz, repeated 200 times at 5 Hz) after what the subject kept relaxed again for 10 min. Then, a second cTBS was applied over M1 followed by 15 min rest. During the last 15 min of the protocol, the triple stimulation protocol with TMS over PMv was tested (3 acquisitions with TMS delivered 5, 7 or 9 ms after median), and the size of ECR MEP was checked. (C), The amplitude of ECR MEP, normalized to its amplitude before cTBS (line at 100% indicated the baseline), is plotted against the time (min) before and after the application of double cTBS. Vertical error bars are ± 1 SEM. ECR, Extensor carpi radialis; EMG, electromyogram; TMS, transcranial magnetic stimulation; cTBS, continuous theta burst stimulation; PMv, ventral premotor area; AMT, active motor threshold.

This protocol was the study's main protocol, which was developed to investigate the putative convergence of peripheral and corticospinal inputs at the level of propriospinal neurons. For this, it was necessary to compare the effects produced by separate inputs and the effects produced by their combination according to the technique of spatial facilitation (Eccles and Lundberg 1957; Pauvert et al. 1998). Therefore, the test response was a reflex response produced in ECR EMG, which was used to quantify the effects of each conditioning situation on motoneuron excitability. There were three conditioning situations: isolated median nerve stimulation, isolated TMS and combined median nerve stimuli and TMS. Seventeen subjects were tested (including the six subjects enrolled in the preliminary experiments; 11 females, age range 22–44 years old, mean age 28.1 ± 1.6 years old); all were right‐handed (Oldfield 1971). The protocol included two experiments: one during which the conditioning TMS was delivered over M1 (at the optimal site for ECR MEP) or on PMv during the other one (1 week apart; TMS site randomly determined). The intensity of conditioning TMS, whatever the stimulation site, was 0.95 × AMT (for ECR MEP), and it was delivered alone or combined to conditioning median nerve stimuli (2 × MT). The effects of conditioning stimuli on ECR motoneurons were evaluated by investigating the changes in H or T reflex (test stimuli). Indeed, because a sizeable H reflex could only be evoked in only five subjects, tendon tap was used in the 12 other subjects to investigate the change in T reflex. Each experiment started by testing the effect of median nerve stimuli on ECR reflexes in order to determine the optimal ISI for producing propriospinal inhibition in ECR motoneurons (Lourenço et al. 2007b); 20 reflexes elicited alone and 20 reflexes conditioned by median nerve stimuli were randomly alternated (0.3 Hz). Then, the effects of combined TMS and median nerve stimuli were tested on ECR reflexes (triple stimulation protocol): 20 reflexes delivered alone were randomly alternated (0.3 Hz) with 60 reflexes conditioned by isolated TMS (over M1 or PMv; *N* = 20), or by isolated median nerve stimuli (*N* = 20) or by combined TMS and median nerve stimuli (*N* = 20). The ISI between median and radial nerve stimuli or tendon tap (both producing ECR reflexes) was fixed and determined in the first part of the protocol. The ISI between median nerve stimuli and TMS was set at 5 ms, 7 ms, and 9 ms according to the time course of the median‐induced ECR MEP suppression investigated during the preliminary experiments (Fig. 1A); the three ISIs were tested in all subjects (three acquisitions with ISI randomly determined), and for the two TMS sites (M1 and PMv).

#### Effect of PMv stimulation on median‐induced inhibition after cTBS over M1

The last series of experiments was performed to further investigate the origin of the corticospinal control from PMv. In the group of 17 subjects, we have selected only the subjects with low AMT to make cTBS comfortable, but four have refused. Therefore, the experiments could only be performed in six subjects (four females, age range 23–39 years old, mean age 29.7 ± 2.2 years old). The experimental design is illustrated in Figure 1B. First, TMS over M1 was adjusted at 1.5 × AMT to produce MEP in ECR EMG during tonic contraction with EMG <5% EMG at MVC (*N* = 15, two sessions of recordings). Then, the subjects stayed relaxed for 15 minutes. Next, the first session of cTBS (TMS at 0.7 × AMT, 3 TMS pulses delivered at 50 Hz and repeated 200 times at 5‐Hz rate frequency) was applied over M1, at the optimal site for MEP in ECR EMG. Then, the subjects stayed relaxed for 10 min again, before they received the second cTBS in order to improve the duration of the neuroplasticity (Goldsworthy et al. 2012). Afterwards, the subjects stayed relaxed for 15 min before the triple stimulation protocol, with TMS over PMv (see above). The full protocol ended with control measures of ECR MEP with TMS over M1 at 1.5 × AMT during tonic contraction with ECR EMG <5% EMG at MVC (*N* = 2 × 15). Care was taken that the time for testing the triple stimulation protocol and MEP in ECR did not exceed 30 min after the second cTBS. Indeed, to develop this protocol, we first tested the effect of spaced cTBS on ECR MEP produced during contraction in 3 subjects, to determine the duration of MEP depression. Figure 1C illustrates the results in 1 subject in whom the MEP was depressed between 15 and 30 min after the second cTBS.

### Analysis

#### Data processing

The change in conditioned ECR MEP after median nerve stimuli was evaluated by calculating the area of rectified ECR EMG within a window of analysis delineated by the beginning of ECR MEP and its duration. The area of test (produced by TMS over M1 alone) and conditioned MEP was evaluated within the same window of analysis, and conditioned MEP was normalized to the mean area of the corresponding test MEP. The peak‐to‐peak amplitude of test MEP in raw ECR EMG was also measured and normalized to Mmax in ECR. The peak‐to‐peak amplitude of ECR MEPs investigated during the cTBS protocol were normalized to the mean peak‐to‐peak amplitude of ECR MEP evoked before cTBS.

The size of test and conditioned reflexes investigated with the triple stimulation protocol and the cTBS protocol was assessed by measuring their peak‐to‐peak amplitude. The conditioned reflexes were normalized to the mean size of the corresponding test reflexes. The size of the latter was normalized to Mmax in ECR.

The difference between test and conditioned responses was used to assess the level of spinal inhibition. The difference between the effects produced on combined stimuli and the algebraic sum of the effects produced by separate stimuli (theoretical effect of combined stimulation) was calculated to quantify the extra effect produced by combined stimuli. Mean values are indicated ± 1 standard error of the mean (SEM).

#### Statistical analysis

The statistical analysis was conducted using SigmaPlot (Systat Software, Inc, San Jose, California) and the significance level was set at *P‐*value <0.05. This software tests automatically the normality (Shapiro–Wilk test) and the homoscedasticity (Levene test) to check the conditions for running parametric tests. If the tests did not pass, we ran nonparametric tests.

**Figure 2:**
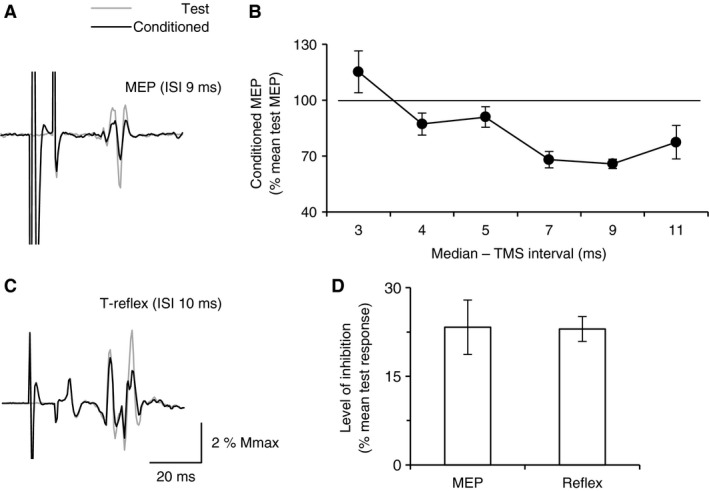
Median‐induced suppression of ECR MEP and reflex. (A), mean test MEP produced by isolated TMS over M1 at 88% MSO (thin line) and mean conditioned MEP (thick line) by median nerve stimulation (2 × MT) delivered 9 ms before TMS in one subject; test and conditioned MEP were normalized to Mmax. (B), mean conditioned MEP (normalized to the corresponding mean test MEP) plotted against the ISI (ms) between median nerve stimuli and TMS over M1, in the same subject as in (A). (C), mean test T reflex produced by isolated tendon tap (thin line) and mean conditioned T reflex (thick line) by median nerve stimulation (2 × MT) delivered 10 ms before tendon tap in the same subject as in (A) and (B); test and conditioned MEP were normalized to Mmax. (D), The mean level of inhibition (difference between test and conditioned responses, expressed as a % mean test responses) observed in the group of 6 subjects when test response was MEP (left column) or reflex (right column). Vertical bars are ± 1 SEM. ECR, Extensor carpi radialis; MEP, motor evoked potential; TMS, transcranial magnetic stimulation; MSO, maximal stimulator output; MT, motor threshold; ISI, inter stimulus interval.

First, we have tested the effect of median nerve stimuli on the size of ECR MEP that we have compared to those on spinal reflex in the same group of subjects. Given the influence of the test response size on the effects of conditioning stimuli due to the heterogeneity of spinal motoneurons within the pool (Crone et al. 1990), we checked whether the size of the test responses was similar in the group using paired *t* test (test MEP % Mmax vs. test reflex % Mmax). Then, we performed a two‐way analysis of variance (ANOVA; response [MEP, reflex] × situation [test, conditioned]) to determine whether the level of median‐induced inhibition was comparable whatever the origin of the test response (Fig. 2D).

The grouped data analysis for the triple stimulation protocol first included Mann–Whitney rank sum test to compare the optimal ISIs between TMS sites (optimal ISI with TMS over M1 vs. optimal ISI with TMS over PMv). Then, the reflex size was compared according to the situation (test vs. median vs. TMS vs. combined) and the TMS site (M1 vs. PMv) at the optimal ISI using two‐way ANOVA (Fig. 4A). We also performed ANOVA for comparing the algebraic sum of the effects of separate stimuli and of combined stimuli (Fig. 4B, C). In this group of data, we also verified that the size of the test reflex % Mmax was similar across the experiments (experiment with TMS over M1 vs. TMS over PMv) using a Mann–Whitney rank sum test. We also compared the latency of T and H reflexes using paired *t* test, which gives support to the ISIs used for median nerve stimuli.

Last, for the cTBS protocol, we compared the size of ECR MEP before and after cTBS over M1 using paired *t* test (MEP amplitude before vs. after cTBS). Then, nonparametric one‐way ANOVA on ranks (Kruskal–Wallis test) was performed to compare the size of the reflex according to the situation (test vs. median vs. TMS vs. combined) and the application of cTBS over M1 (before vs. after cTBS). We also compared the level of extra‐inhibition produced on combined stimuli using paired *t* test.

When ANOVAs were significant, multiple pairwise comparisons were performed using post hoc tests (Holm–Sidak and Tukey tests).

## Results

### Median‐induced suppression of ECR MEP and reflex

**Figure 3:**
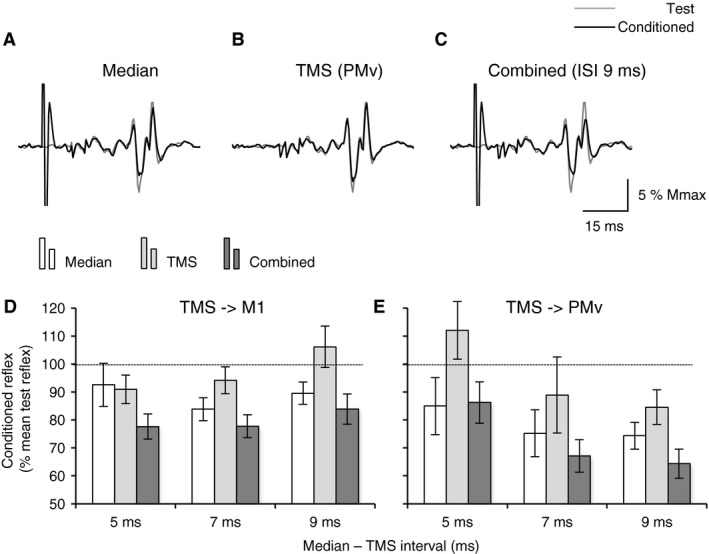
Results of triple stimulation protocol in one subject. (A–C), mean test T reflex (% Mmax) produced in ECR EMG after tendon tap (thin line) and mean conditioned reflex (thick line) by isolated median nerve stimulation (2 × MT, ISI 11 ms; A) or by TMS over PMv (0.95 × AMT; B) or by combined median nerve stimulation and TMS over PMv (ISI 9 ms; C). DE, mean conditioned reflexes (% mean test reflex) in the same subject, observed after isolated median nerve stimuli (white columns), or after isolated TMS (light gray columns) over M1 (D) or over PMv (E), or after combined stimuli (dark gray columns). Vertical bars are ± 1 SEM. ECR, Extensor carpi radialis; EMG, electromyogram; MT, motor threshold; ISI, inter stimulus interval; TMS, transcranial magnetic stimulation; AMT, active motor threshold; PMv, ventral premotor area.

Figure 2A shows that ECR MEP was smaller when TMS over M1 was preceded by median nerve stimulation (ISI 9 ms), compared to MEP produced by isolated TMS. In the same subject, the full‐time course of the median‐induced MEP suppression is illustrated in Figure 2B, showing MEP inhibition at ISIs 7, 9 and 11 ms. The same experiment was performed in 6 subjects and such a median‐induced MEP suppression was observed in all the 6 subjects. On average, the minimal ISI for producing MEP suppression was 6.0 ± 0.8 ms and maximal inhibition was observed at 7.8 ± 1.0 ms. Within the group, the minimal ISI for MEP inhibition was 4 ms, and the maximal ISI was 11 ms. In all the 6 subjects, the median‐induced MEP inhibition was observed at ISIs 7 and 9 ms; in half, MEP inhibition was also observed at ISIs 5 ms. These results were used to select the ISIs between median nerve stimulation and TMS in the triple stimulation protocol (Fig. 3D, E).

In the same group of 6 subjects, we compared the level of median‐induced inhibition of ECR MEP and reflex (T reflex in 5 subjects and H reflex in 1 subject). Figure 2C illustrates in the same subject as in Figure 2A, the smaller reflex produced by combined stimuli with 10 ms ISI (median nerve stimulation preceding tendon tap). In the group, the mean test size of MEP and reflexes was similar (9.0 ± 2.0% Mmax for MEP vs. 8.9 ± 1.3% for the reflex; paired *t* test, *t* = 0.0241, 9 degrees of freedom (df), *P *=* *0.9). On average, the level of inhibition was similar whatever the origin of the test response: 23.3 ± 4.6 versus 23.0 ± 2.1% mean test response, for MEP and reflex, respectively (Fig. 2D; two‐way ANOVA: response [MEP vs. reflex: *F* = 0.0281, 1 df, *P *=* *0.8], situation [test vs. conditioned: *F* = 1.714, 1 df, *P *=* *0.2], response × situation: *F* = 0.0179, 1 df, *P *=* *0.8).

### Effects of combined median nerve stimuli and TMS over M1 and PMv on ECR reflex

Figure 3A, C show in one subject that the T reflex in ECR EMG was smaller when conditioned by isolated median nerve stimuli (A; ISI 10 ms) and combined stimuli (C; median nerve + TMS over PMv with 9‐ms ISI). On the contrary, isolated TMS over PMv hardly modify reflex amplitude (B). Figure 3D, E illustrate the mean conditioned T reflexes in the same subject as in Fig. 3A‐C, after isolated median nerve stimuli, isolated TMS or combined stimuli. The reflex depression appeared stronger on combined stimuli, whatever the TMS site.

**Figure 4:**
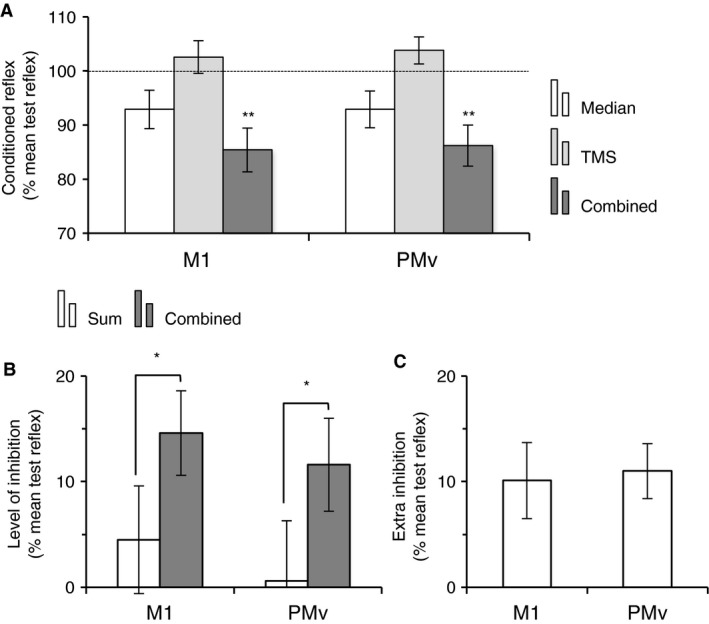
Comparison of algebraic sum of separate stimuli and the effects of combined stimuli in the group of 17 subjects. (A), mean conditioned reflexes (% mean test reflex) after median nerve stimuli (white columns), after isolated TMS (gray light columns) and after combined stimuli (dark gray columns), when TMS was applied over M1 (left columns) or over PMv (right columns). Horizontal dotted line shows the 100% level, indicating no effect of conditioning. (B), level of inhibition (% mean test reflex) estimated by calculating the algebraic sum of the effects produced by separate stimuli (white columns) and that produced on combined stimuli (dark gray columns) when TMS was over M1 (left columns) or over PMv (right columns). (C), difference between inhibitions on combined stimuli and the algebraic sum (% mean test reflex), when TMS was over M1 (left column) or over PMv (right column). Vertical bars are ± 1 SEM. *P <* *0.05 and **P <* *0.01. TMS, transcranial magnetic stimulation; PMv, ventral premotor area. TMS, transcranial magnetic stimulation; PMv, ventral premotor area.

Suppression of ECR reflexes on combined stimuli has been observed in all the 17 subjects; at least for 1 of the motor areas stimulated in 7 subjects (M1 for 3/7 and PMv for 4/7), and for the 2 motor areas in the 10 remaining subjects. As observed in the subject illustrated in Figure 3D, E, the effects could vary according to the ISI. Therefore, for each individual, we retained for grouped analysis the ISI at which the inhibition on combined stimuli was the strongest (optimal ISI). We found that the mean optimal ISI was similar for M1 and PMv (7.0 ± 0.4 vs. 6.6 ± 0.4 ms, respectively; Mann–Whitney rank sum test, *U* = 122.50, *T* = 319.50, *P *=* *0.4); reflex inhibition was observed at similar ISI in 6/17 subjects, at longer or shorter ISIs for M1 in 5/17 and 6/17 subjects, respectively. Two‐way ANOVA was run to compare the reflex in each situation (test vs. median vs. TMS vs. combined) and TMS site (M1 vs. PMv; Fig. 4A). The size of the reflex significantly changed according to the situation (*F* = 13.562, 3 df, *P *<* *0.001) but there was no difference between TMS site (*F* = 0.0589, 1 df, *P *=* *0.8) and there was no interaction between factors (situation × TMS site, *F* = 0.0187, 1 df, *P *=* *0.9). Post hoc analyses (Holm–Sidak) revealed that the reflex suppression reached the statistical significant level on combined stimuli only (*t* > 3.283, *P *<* *0.01 for both TMS sites). Whatever the TMS site, isolated TMS did not influence the reflex size (*t* < 0.893, 0.5 < *P *<* *0.8), and the weak median‐induced reflex suppression was not significant (*t* > 1.677, 0.1 < *P *<* *0.2).

Conventionally, the effects of combined stimuli are compared to the algebraic sum of the effects produced by separate stimuli (Eccles and Lundberg 1957; Pauvert et al. 1998; Pierrot‐Deseilligny and Burke 2012). Figure 4B shows the algebraic sum of the effects produced by isolated median nerve stimuli and TMS and the effects produced on combined stimuli. Two‐way ANOVA revealed significant difference between the levels of inhibition (sum vs. combined, *F* = 4.732, 1 df, *P *<* *0.05) but no difference between the TMS sites (*F* = 0.511, 1 df, M1 vs. PMv, *P *=* *0.4) and no interaction between factors, which further supports that the inhibition was stronger on combined stimuli, without difference between the TMS sites (compare the levels of extra‐inhibition on combined stimuli between cortical areas in Fig. 4C).

It has to be noted that the size of the test reflex responses was similar during the 2 experiments: 9.9 ± 2.1% Mmax during the experiment with TMS over M1 vs. 8.2 ± 0.9% during the experiment with TMS over PMv (Mann–Whitney rank sum test, *U* = 117.0, *T* = 243.0, *P *=* *0.9). We also observed no significant difference in the latency of H and T reflexes (19.2 ± 1.1 vs. 19.7 ± 0.6 ms, respectively; *t* test, *t* = 0.396, 15 df, *P *=* *0.69). Accordingly, the mean ISI between median nerve and radial nerve stimuli or tendon tap (evoking H and T reflex in ECR EMG, respectively) was similar (11.6 ± 0.5 ms, on average), ranging between 10 ms and 14 ms.

### Effects of TMS over PMv after cTBS over M1

**Figure 5:**
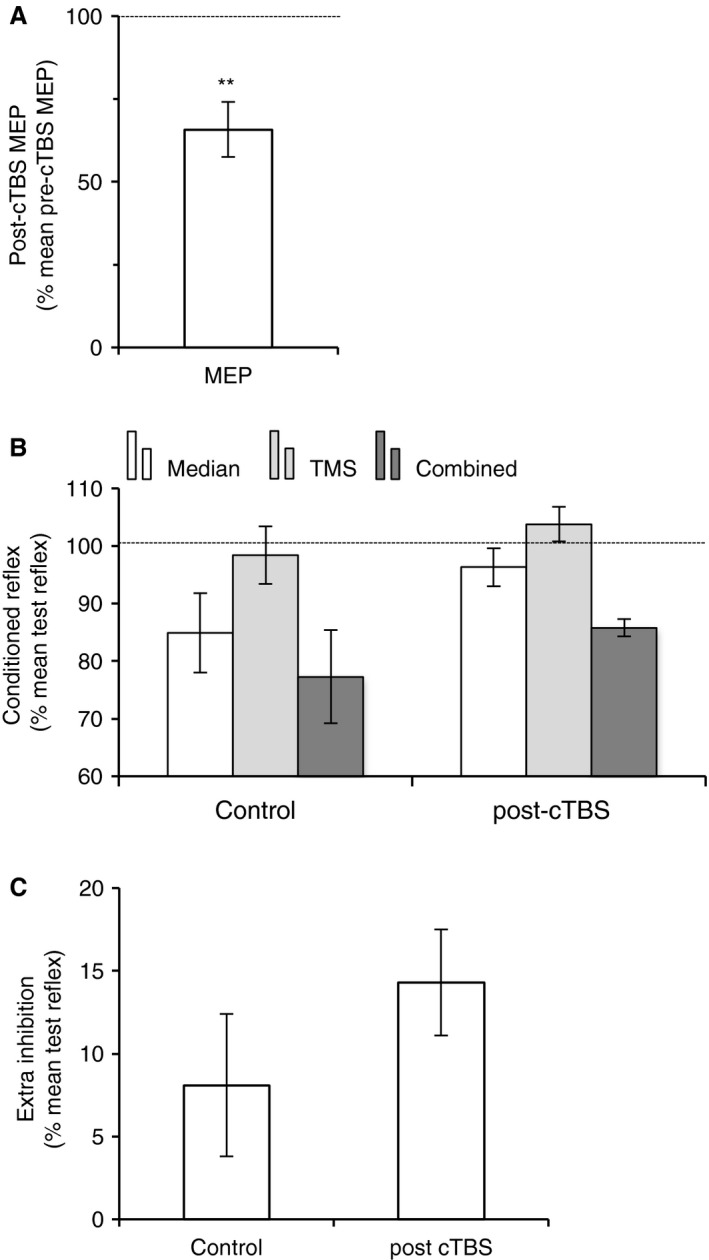
Effect of cTBS over M1 on the extra‐inhibition produced by TMS over PMv. (A), mean amplitude of MEP produced in ECR EMG after TMS over M1 (TMS intensity at 1.5 × AMT), expressed as a % of the mean MEP amplitude before cTBS over M1, in the group of six subjects. Horizontal dotted line shows the 100% level, indicating no change in MEP size. (B), mean difference (the six subjects as in (A)) between the effects of combined stimuli (with TMS over PMv) and the algebraic sum of separate stimuli (extra inhibition), normalized to the mean size of the test reflex produced in ECR EMG. The extra inhibition produced without cTBS over M1 is indicated by the left column, and the extra‐inhibition after cTBS over M1, by the right column. Vertical bars are ± 1 SEM. **P <* *0.01. ECR, Extensor carpi radialis; EMG, electromyogram; TMS, transcranial magnetic stimulation; cTBS, continuous theta burst stimulation; PMv, ventral premotor area; AMT, active motor threshold.

Double cTBS was applied over M1 in order to depress the cortical excitability at this level. Accordingly, the MEP produced in ECR EMG no later than 30 min after cTBS was significantly depressed in all the 6 subjects, compared to its baseline before cTBS. Figure 5A shows the mean amplitude of ECR MEP after cTBS in the group, normalized to its amplitude before the double cTBS (paired *t* test, *t* = 6.468, 5 df, *P *<* *0.01).

**Figure 6:**
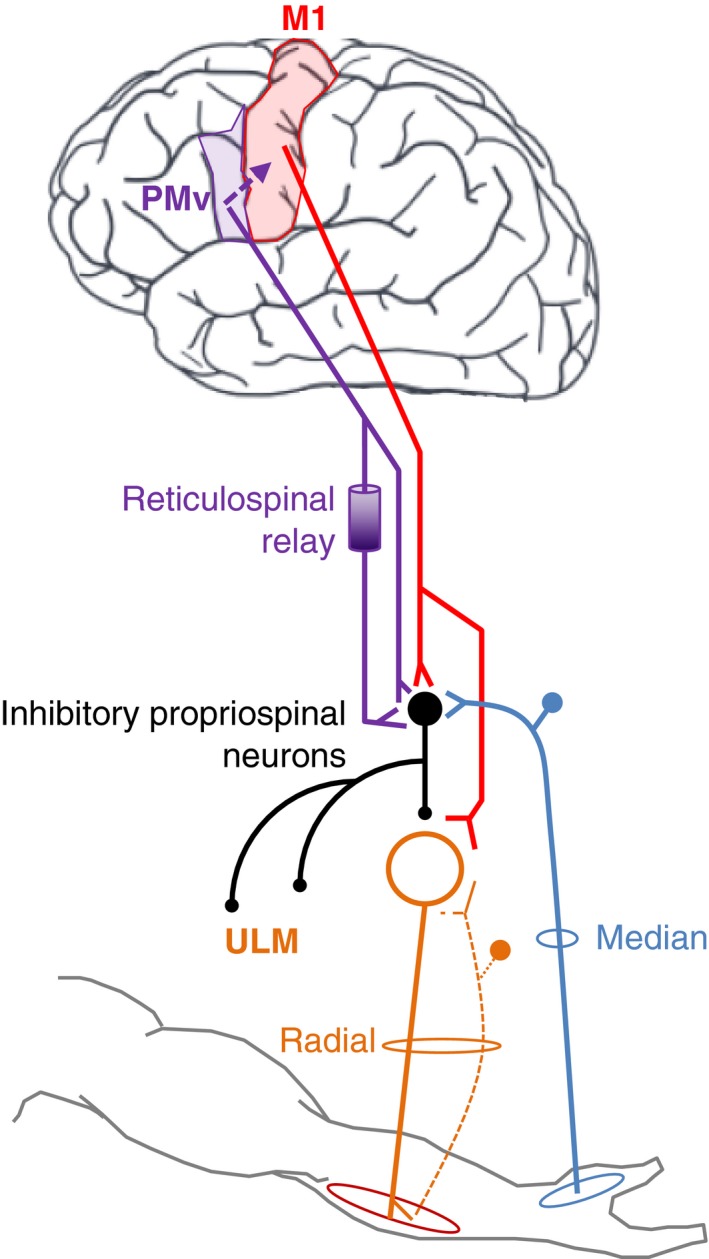
Hypothetic diagram of the corticospinal connectivity. The descending projections from M1 (red) project directly onto ECR motoneurons (orange circle and line) and on inhibitory propriospinal neurons (PN; black filled circles and black line) which project onto ECR motoneurons too, and other upper limb motoneurons (ULM). Similarly, corticospinal projections from PMv (purple) facilitate the inhibitory propriospinal transmissions to ECR motoneurons, directly and/or through reticulospinal projections via relay at brainstem level. The inhibitory propriospinal also receive afferent inputs from median nerve (blue). Muscle spindle group Ia afferents (orange dotted line) running into the radial nerve (as ECR motor axons) mediate monosynaptic excitation to ECR motoneurons producing H‐ and T‐reflex in ECR EMG. ECR, Extensor carpi radialis; EMG, electromyogram; PMv, ventral premotor area; PN, propriospinal neurons; ULM, upper limb motoneurons.

The triple stimulation protocol, with TMS over PMv, was tested after double cTBS over M1. Figure 5B shows the mean size of conditioned reflexes before cTBS (control) and after cTBS over M1. Given the significant *P* value of the Kruskal–Wallis test on the grouped data (reflex size in all situation [test vs. median vs. TMS vs. combined], before and after cTBS; *H* = 24.460, 7 df, *P *<* *0.001), we performed post hoc analyses for pairwise comparisons using Tukey test, which revealed no significant difference in reflex size (test and conditioned by median or TMS or combined stimuli, 0 < q < 1.691, *P *>* *0.05) before and after cTBS. In none subject, the inhibition was less after cTBS; the extra‐inhibition was similar in 1/6 and stronger in 5/6 subjects after cTBS. Figure 5C shows the extra‐inhibition produced on combined stimuli, which was similar before (control) and after cTBS (paired *t* test, *t* = −1.552, 5 df, *P *=* *0.1).

## Discussion

This study has shown that median nerve stimulation can suppress ECR MEP to the same extent as ECR reflex, and when combined to subthreshold TMS over M1 or PMv, the reflex inhibition was enhanced. Indeed, the inhibition on combined stimuli was greater than the algebraic sum of the effects produced by separate stimuli. The level of the resulting extra‐inhibition was similar whatever the location of TMS (over M1 or PMv). Last, paired cTBS over M1, which depressed the size of ECR MEP, did not influence the level of extra‐inhibition produced by PMv stimulation.

### Convergence of peripheral and M1 corticospinal volleys onto inhibitory propriospinal neurons

Spinal inhibition with central delay increasing with the rostrocaudal motoneuron location has been observed after peripheral nerve stimulation (Lourenço et al. 2007b), and it has been suggested that this inhibition is likely mediated by inhibitory propriospinal system (Marchand‐Pauvert and Iglesias 2008). This inhibition is particularly depressed in dystonic patients, likely due to disrupted descending control (Lourenço et al. 2007a). This suggests that inhibitory propriospinal neurons may receive corticospinal inputs but this had never been tested so far in humans. In this study, we first tested the possible interaction between corticospinal inputs from M1 and peripheral volleys activating inhibitory propriospinal neurons.

In the group of subjects in whom we tested both MEPs and reflexes, we found that MEP occurred in ECR EMG 2 ms earlier than the reflex. So, TMS‐induced descending inputs required 2 ms less than peripheral inputs (elicited at elbow level for producing H‐reflex) to make motoneuron discharge. On the other hand, the extra peripheral conduction time between wrist and elbow has been estimated to be 4 ms (Marchand‐Pauvert et al. 2000). Therefore, the convergence of peripheral inputs elicited at the wrist level (median nerve stimulation) and corticospinal volleys could be expected at ISI 2 ms (=4 − 2 ms). The median‐induced MEP suppression occurred on average at ISI 6 ms. Therefore, the central delay for inhibition was 4 ms (=6 − 2 ms), as observed previously for the propriospinal inhibitory system using peripheral nerve stimulation only (Lourenço et al. 2007b). Moreover, it is interesting to note that the ISIs for convergence at the level of inhibitory propriospinal neurons was similar as those observed for interaction at the level of excitatory propriospinal neurons (Iglesias et al. 2007). Further, this study has shown that median nerve stimuli suppressed MEPs and reflexes to the same extent, which strongly supports the convergence at interneuron level producing direct inhibition of ECR motoneurons (Marchand‐Pauvert and Iglesias 2008). Indeed, disfacilitation of the descending command through interneurons inhibiting excitatory propriospinal neurons would have influenced MEP size to a greater extent than that of the reflex (Burke et al. 1994). Moreover, our present results further suggest that the median‐induced inhibition is not presynaptic in origin because corticospinal inputs, and thus MEPs, are not sensitive to presynaptic inhibition (Nielsen et al. 1999).

In the triple stimulation protocol, we tested the effects of combined median nerve and corticospinal volleys on the excitability of ECR motoneurons assessed by testing the resulting change in ECR reflexes. The TMS intensity was subthreshold for evoking an MEP in ECR EMG to avoid superimposition of compound potentials (MEP + reflex). However, at 0.95 × AMT, TMS is known to produce descending volleys that can enhance single motor unit discharge (Day et al. 1989; Burke et al. 1993; Di Lazzaro et al. 1998; Lackmy‐Vallee et al. 2012). One would thus expect that TMS had increased reflex size when given alone but this was not the case likely because the ISI was not optimal for the convergence of corticospinal and group Ia volleys at the motoneuron level; the theoretical ISI for this was −2 ms (reflex inputs delivered 2 ms after TMS), and we only tested ISIs 5, 7 and 9 ms to optimize the convergence at the interneuron level. On the other hand, combined stimuli produced stronger inhibition than that we could expect based on the algebraic sum of isolated stimuli. This suggests a spatial facilitation of descending and peripheral inputs at interneuron level leading to stronger inhibition in motoneurons than that produced by separate stimuli (Eccles and Lundberg 1957).

Therefore, the present results further support that inhibitory propriospinal neurons in humans receive corticospinal inputs from M1, which allows a direct descending feed‐forward inhibition of spinal motoneurons.

### Evidence for corticospinal control from both M1 and PMv areas

During the triple stimulation protocol, combined activation of peripheral and descending inputs produced a stronger reflex inhibition than the algebraic sum of effects produced by isolated stimuli whether TMS was delivered over M1 or PMv, and the level of extra‐inhibition was similar whatever the TMS site. This result suggests that subthreshold TMS over PMv induced descending inputs, facilitating the discharge of inhibitory propriospinal neurons, to the same extent as the descending inputs produced by subthreshold TMS over M1.

In macaques, although the median conduction velocity (CV) of PTNs in PMv is slower (20 m/s) than those in M1 (30 m/s), there is a great overlap (Firmin et al. 2014; Kraskov et al. 2014). Transposing this data to humans, there is possibility that putative descending inputs from PMv reach spinal neurons at the same time than those from M1 (manifesting at similar ISI). However, according to the mean CV, one would expect that PMv stimulation would have produced reflex extra‐inhibition at longer ISIs than after M1 stimulation. According to the mean distance between vertex point and C7 vertebra (0.25 m), the extra time after PMv stimulation can be estimated to be at ~4 ms, so 4‐ms longer ISI for PMv stimulations than for M1. However, a recent study has reported only 1.1‐ms difference between MEPs produced by stimulations applied over M1 and premotor areas (Fornia et al. 2016). In this study, the ISI for extra‐inhibition was longer for PMv in only 5/17 subjects. The discrepancy between experimental results and theoretical estimation can be explained by the well‐known desynchronization of corticospinal descending volleys produced by TMS over M1. This would be likely the case when stimulating PMv as well, which strongly limits the time resolution of the method. Moreover, for a better estimation of the timing, we should have measured the distance in each subject and we should have tested more ISIs, with 0.5–1‐ms difference to evaluate the shorter ISI for convergence, but this would have strongly slowed down the protocol. Last, peripheral inputs can interact with successive descending volleys, which results in temporal summation occurring at successive ISIs (Pierrot‐Deseilligny and Burke 2012). When developing the protocol, we assumed that it would be very difficult to determine the exact timing of spatial summation of peripheral inputs with descending volleys from M1 or PMv, and that the time resolution of the method would not allow distinguishing small differences. Therefore, we chose to investigate the same ISIs for both TMS sites; those we found optimal for M1 with the possibility that they were not optimal for PMv. Moreover, the ISIs 5, 7 and 9 ms did not correspond to the shorter ISI for summation but those at which the median‐induced inhibition was the strongest and at which it has been observed in all the subjects tested in the preliminary experiments.

The conduction time between M1 and PMv has been estimated to be 1–2 ms in macaques (Godschalk et al., 1984). Using paired TMS in humans, the ISIs for interaction between PMv and M1 was found longer (6–8 ms) due to anatomical differences and complex neural processing (Davare et al. 2008; Bäumer et al. 2009). Therefore, since we found extra‐inhibition at similar ISIs for both M1 and PMv, a common descending influence from PMv and M1, through PMv‐M1 interaction, seems rather unlikely. We therefore assume that the descending control from PMv might be mediated through its own pathway, independent of M1. However, due to the proximity of both areas and the poor spatial selectivity of TMS, it can be argued that the same PTNs in M1 were activated whatever the TMS site. However, this hypothesis is rather unlikely due to the low TMS intensity we used (Siebner et al. 2009).

Another issue when stimulating PMv is the difficulty to produce direct motor response, putting some doubts on the efficacy of TMS to activate axons at this level. To get rid of this, we used a conditioning TMS intensity quite in line with those used to investigate the interactions between PMv and M1 when testing paired‐pulse TMS (Davare et al. 2008, 2009, 2010; Bäumer et al. 2009; de Beukelaar et al. 2016). Moreover, since TMS at 0.95 × AMT over M1 can produce descending volleys (Day et al. 1989), we presumed that it could also induce descending volleys when applied over PMv. Indeed, it has been shown in macaques that PMv has descending projections reaching directly the cervical cord (Dum and Strick 1991; He et al. 1993; Borra et al. 2010), or through the bulbar reticular formation (Borra et al. 2010). In humans, evidences for PTNs in PMv arose from a study using diffusion‐weighted imaging and probabilistic tractography showing descending fibers with trajectory somewhat different to that described in macaques (Newton et al. 2006; Verstynen et al. 2011). Moreover, the role of PMv in motor recovery after stroke likely involved descending inputs from PMv or mediated through reticular formation (Benecke et al. 1991; Kantak et al. 2012). Therefore, we assume that the reinforcement of reflex inhibition after PMv stimulation observed in this study, might be mediated through direct corticopinal pathway to inhibitory propriospinal neurons or through reticulospinal inputs (Illert et al. 1977). To further confirm this hypothesis, the triple stimulation protocol with TMS over PMv was tested after cTBS‐induced reversible disruption of M1. Indeed, if the corticospinal control observed after PMv stimulation were due to PMv‐M1 interaction, reflex extra‐inhibition would have been less after cTBS over M1, as much as observed when assessing the MEP size. We did not find any significant change in the level of extra‐inhibition, and if anything, it was even stronger after cTBS over M1. This further suggests that descending command activated by TMS over PMv had interacted with cervical inhibitory propriospinal neurons to enhance reflex inhibition, without passing through M1. This command was likely mediated by PMv corticospinal projections to upper cervical segments and/or through the putative projections to reticulospinal pathway.

In summary, we have brought evidences regarding the existence of neural connectivity between PMv and cervical inhibitory propriospinal neurons. We also propose that this connectivity is not limited to PMv‐M1 interaction, but it likely includes descending command mediating inhibition to cervical motoneurons through propriospinal neurons.

### Functional significance

Several points of convergence led us to hypothesize on the connectivity between the premotor cortex and the C3–C4 propriospinal neurons. First, the corticospinal projections from PMv can reach cervical motoneurons but the majority of PTNs ends at the upper cervical levels, where propriospinal neurons are located (Dum and Strick 1991; He et al. 1993; Borra et al. 2010). In addition, premotor cortex and cervical propriospinal system are both particularly involved in visually‐guided movements including target reaching and grasping (Iglesias et al. 2007; Koch and Rothwell 2009; Giboin et al. 2012; Isa et al. 2013; Rizzolatti et al. 2014). Last, the depression of spinal excitability during action observation, which is accompanied by the inactivation of only 1/3 mirror neurons in PMv and M1 (Kraskov et al. 2009, 2014), might be mediated by direct PMv corticospinal projections, or through reticular formation (Baldissera et al. 2001; Kraskov et al. 2009). This study shows that corticospinal projections from M1 and PMv can strengthen the diffuse spinal inhibition mediated by inhibitory propriospinal neurons. In addition to the brain mechanisms, such a corticospinal connectivity can participate in focusing the motor command on the relevant spinal motoneurons during visually guided reach‐to‐grasp movements and to prevent unwilling muscle contraction.

## Conclusions

The spatial facilitation method allowed us to demonstrate that M1 and PMv can activate cervical propriospinal neurons that inhibit directly ECR motoneurons in humans. This control from PMv was not altered after paired cTBS over M1, suggesting that the main part of the projections from PMv to propriospinal neurons is not mediated through M1. We propose that cervical inhibitory propriospinal neurons likely mediate diffuse upper limb motoneuron inhibition under the control of M1 and PMv to avoid unwilling muscle activation. Future studies using the present methodology would be interesting to further investigate the role of the M1/PMv‐propriospinal connectivity during motor control and learning, and in patients with movement disorders.

## Conflict of Interest

None declared.
